# Encapsidated Host Factors in Alphavirus Particles Influence Midgut Infection of *Aedes aegypti*

**DOI:** 10.3390/v10050263

**Published:** 2018-05-16

**Authors:** David Mackenzie-Liu, Kevin J. Sokoloski, Sarah Purdy, Richard W. Hardy

**Affiliations:** 1 Department of Biology, Indiana University, Bloomington, IN 47405, USA; dwliu@indiana.edu (D.M.-L.); sjpurdy@umail.iu.edu (S.P.); 2 Department of Microbiology and Immunology, and the Center for Predictive Medicine for Biodefense and Emerging Infectious Diseases, University of Louisville School of Medicine, Louisville, KY 40202, USA; kevin.sokoloski@louisville.edu

**Keywords:** alphavirus, mosquito, transmission

## Abstract

Transmission of mosquito-borne viruses requires the efficient infection of both a permissive vertebrate host and a competent mosquito vector. The infectivity of Sindbis virus (SINV), the type species of the Alphavirus genus, is influenced by both the original and new host cell. We have shown that infection of vertebrate cells by SINV, chikungunya virus (CHIKV), and Ross River virus (RRV) produces two subpopulations of virus particles separable based on density. In contrast, a single population of viral particles is produced by mosquito cells. Previous studies demonstrated that the denser vertebrate-derived particles and the mosquito-derived particles contain components of the small subunit of the host cell ribosome, whereas the less dense vertebrate-derived particles do not. Infection of mice with RRV showed that both particle subpopulations are produced in an infected vertebrate, but in a tissue specific manner with serum containing only the less dense version of the virus particles. Previous infectivity studies using SINV particles have shown that the denser particles (SINV^Heavy^) and mosquito derived particles SINV^C6/36^ are significantly more infectious in vertebrate cells than the less dense vertebrate derived particles (SINV^Light^). The current study shows that SINV^Light^ particles, initiate the infection of the mosquito midgut more efficiently than SINV^Heavy^ particles and that this enhanced infectivity is associated with an exacerbated immune response to SINV^Light^ infection in midgut tissues. The enhanced infection of SINV^Light^ is specific to the midgut as intrathoracically injected virus do not exhibit the same fitness advantage. Together, our data indicate a biologically significant role for the SINV^Light^ subpopulation in the efficient transmission from infected vertebrates to the mosquito vector.

## 1. Introduction

Arthropod-borne viruses (arboviruses), such as Alphaviruses and Flaviviruses, continue to be a global health challenge, and recent outbreaks in Europe, Asia, and the Americas highlight their significance [1,2,3,4,5]. Alphaviruses, such as the type species Sindbis (SINV) and Chikungunya (CHIKV), belong to the family Togaviridae, and are enveloped, positive-sense, single-stranded RNA viruses with a genome of approximately 12 kb in length. The enzootic alphavirus transmission cycle consists of a series of step-wise events alternating between invertebrate and vertebrate hosts. Initially, for the virus to persist in nature, it must infect a competent mosquito vector via an infectious blood meal. The virus must replicate within the midgut prior to disseminating to the salivary glands thus enabling transmission to the next vertebrate host during successive blood feeding [6]. The virus must then replicate efficiently in the new vertebrate host to attain sufficiently high titers before being transmitted to a naïve mosquito host during a blood meal [7]. This transmission cycle has been the focus of intense study and is influenced by many host and viral factors. Here, we examine a new viral determinant of transmission from the vertebrate host to the mosquito.

Transmission of arboviruses is dependent on the virus overcoming several barriers. In the mosquito, there are four major checkpoints that the virus must overcome for transmission to occur; namely, the midgut infection and escape barriers, and the salivary gland infection and escape barriers [8,9,10,11,12,13]. Infection barriers can be influenced by many factors, including polymorphisms in the viral glycoproteins and nonstructural proteins, the genotypic pairing of the mosquito and virus isolate, physical environment of the mosquito, and the microbiome composition of the midgut including the presence or absence of the intracellular bacterium Wolbachia [14,15,16,17,18,19]. After feeding on a vertebrate, the blood meal moves down to the mosquito midgut where virus particles must contact epithelial cells before further digestion of the blood meal takes place [20]. Following the successful infection of the midgut, the virus must overcome midgut escape barriers to disseminate to other tissues such as the salivary glands [8,21,22].

A potentially important determinant of midgut infection is the glycosylation of the alphavirus glycoproteins [23]. Alphaviruses exhibit glycosylation patterns unique to the propagating host cell type. Viral particles derived from insect cells have high-mannose glycan additions to their surface glycoproteins, whereas vertebrate derived particles tend to have different complex glycan moieties [24,25]. Mutating glycosylation sites has been shown to significantly affect infection in vertebrate and insect cells. Specifically, mutating residue 245 on E1 showed a replication deficiency in both cell culture and live mosquitoes [26]. Hence, N-linked glycans may play a significant role in the efficiency with which mosquitoes become infected and the virus disseminates.

In addition to various glycosylation patterns, we have also found that the density of alphavirus particles varies in a propagating host cell-dependent manner [27]. As previous studies have shown, infectious virus particles derived from mosquitoes exhibit uniform density, whereas particles derived from vertebrate cells may be segregated into two different infectious populations based on density. These populations were named “light” and “heavy” particles, based on their relative density. Electron microscopy demonstrated that regardless of the host type, or particle density, all Sindbis virus (SINV) particles had the same volume and morphology. Altered particle density was determined to be due to the incorporation of host derived ribosomal components (HDRCs), and the inclusion of the HDRCs is correlated with increased infectivity in vertebrate cell lines [27]. In the current study, we extended our analyses of alphavirus particle densities from cell culture systems to animal models of infection, including both vertebrate and mosquito host models. The data presented here indicated that the differences in particle density is recapitulated in infected animals. These observations raised the question of how the different viral subpopulations may impact the transmission cycle of alphaviruses. Previous work has shown mosquito cell-derived virus to be highly infectious in vertebrate cells [28]. As only “heavy” particles are produced by mosquitoes the host is inoculated with a single population of virus particles following the infectious blood meal. Subsequently, the virus will amplify in the vertebrate host producing two populations of particles with a heavy bias toward the production of light particles, hence a naïve mosquito will feed and be inoculated with light particles. Our previous work focused on the infectivity of different virus particle populations in vertebrate cells, and we had found that light particles had a very high particle:plaque forming unit ratio indicating low infectivity [27]. Therefore, the biological significance of the light particle production in vertebrate cells remains unclear. In this study, we have examined the role of vertebrate cell-derived subpopulations during the infection of mosquitoes via blood meal. We have found that light particles infect mosquito midgut tissues more efficiently than heavy particles leading to more efficient dissemination of virus within the insect. These data indicate that the internal cargo of the virus particle, beyond the virus genome, is an important determinant of viral transmission.

## 2. Materials and Methods

### 2.1. Cell Lines

BHK-21, Vero, and C6/36 cells were cultured in minimal essential medium (MEM) supplemented with 10% FBS, nonessential amino acids, L-glutamine, and 1× antibiotic-antimycotic solution (Corning, Corning, NY, USA). BHK-21 and Vero cells were maintained at 37 °C in the presence of 5% CO_2_, and C6/36 cells were maintained at 28 °C in the presence of 5% CO_2_.

### 2.2. Virus Preparation and Purification

CHIKV 181/25, Ross River virus (RRV) T48, and SINV MRE3’2J-GFP were prepared by transfecting BHK-21 cells with in vitro transcribed RNA with Lipofectamine LTX (Sigma Aldrich, St. Louis, MO, USA) [29,30,31,32]. The culture supernatant was then collected and clarified by centrifugation and stored at −80 °C. Titer of the virus was determined on BHK-21 cells. Virus particles were then further purified as previously described [27]. Briefly, confluent BHK-21 cells in five 150 mm dishes were infected with SINV at a multiplicity of infection (MOI) of 3 PFU/cell. The culture supernatant was then harvested 18 h post-infection. Virus particles were then concentrated by pelleting through a 27% sucrose cushion prepared in HNE buffer (20 mM HEPES (pH 7.4), 150 mM NaCl, 5 mM EDTA) by centrifugation at 185,000× *g* for 2 h in a 60 Ti rotor (Beckman-Coulter, Brea, CA, USA). The pelleted particles were then suspended in HNE buffer before being added to a linear gradient of 15 to 45% (mass/vol) sucrose in HNE. The linear gradients were prepared with a Gradient Master Apparatus (BioComp Instruments, Fredricton, NB, Canada) by using the preprogrammed settings. Virus particles were banded by centrifugation at 250,000× *g* for 3 h in a SW41 rotor (Beckman-Coulter, Brea, CA, USA). The virus subpopulations were collected via needle aspiration and stored at 4 °C for short-term use or at −80 °C.

### 2.3. Virus Particle Quantification

To determine the infectious populations derived from in vivo specimens, tissue samples were taken from mouse quadriceps muscle, serum, and ankle one day post-subcutaneous injection (the known peak of viral titer in vivo) of RRV. The tissue samples, when necessary, were homogenized via bead-beating in 1× PBS. The virus particle containing supernatants were clarified via centrifugation at 16,000× *g* for 10 min at 4 °C; the clarified supernatant was purified as described above. Five to seven-day old mosquitoes were injected with SINV and were homogenized two days post-injection. The various samples were purified and centrifuged as described and were assayed for PFU per gradient fraction to determine infectious densities as previously described [27]. Quantification of virus particle numbers was performed as previously described with minor changes [28]. Briefly, a 5 µL aliquot of purified virus was incubated at 95 °C to release the RNA genome. Then, the released RNA was used as a template for cDNA synthesis via M-MuLV reverse transcriptase (NEB, Ipswich, MA, USA) with random hexamer primers (IDT, Coralville, IA, USA). The resulting cDNA was then added to a reaction mixture of 2× SensiFast Hi-Rox SYBR (Bioline, London, UK) and 200 nM final concentration nsP1 primers (Table 1). The reaction mixture was then cycled in a StepOnePlus Real-Time PCR System (Applied Biosystems, Waltham, MA, USA). The CT values of virus samples were compared to internal standard curves to determine the number of genome equivalents.

### 2.4. Analysis of Viral Glycoproteins

Purified C6/36 and BHK-21 derived virus particles were subjected to analysis by 12% SDS-PAGE with trichloroethanol for stain-free imaging with a ChemiDoc (BioRad, Hercules, CA, USA). Virus samples were treated with PNGase F (NEB) to remove N-linked oligosaccharides according to the manufacturer’s protocol.

### 2.5. Mosquito Infections

*A. aegypti*, RexD (Puerto Rico) derived Higgs White Eye (HWE) strain, were reared in an insect incubator (Percival Model I-36VL, Perry, IA, USA) at 28 °C and 75% humidity with 12 h light/dark cycle. Five to seven-day old female mosquitoes were injected with 5 × 10^5^ SINV particles via NanoJect II (Drummond, Broomall, PA, USA). Five to seven-day old females were also starved for 24 h and then allowed to feed for 1 h from pre-warmed citrated rabbit blood supplemented 1:4 with 1 × 10^8^ SINV particles. The blood was kept at 37 °C in a water-jacketed glass feeder with a hog intestine barrier. Engorged mosquitoes were then isolated and incubated at 28 °C and provided 10% sucrose. At specified time points, mosquitoes were chilled and dissected and snap frozen in liquid nitrogen before being stored at −80 °C before further processing. Infected mosquitoes were dissected at various time points. Dissected organs were visualized directly via fluorescence microscopy (Olympus, Tokyo, Japan). Samples for qRT-PCR were homogenized in TRIzol (Sigma Aldrich) reagent and further processed for RNA extraction.

### 2.6. Quantitative Analysis of Mosquito Transcripts

TRIzol extracted RNA from mosquito samples were reverse transcribed via M-MuLV (NEB) and 500 ng random hexamer primers (IDT). The resulting cDNA was then added to a reaction mixture of 2× SensiFast Hi-Rox SYBR (Bioline) and 200 nM final concentration primers. The reaction mixture was then cycled in a StepOnePlus Real-Time PCR System (Applied Biosystems) and the resulting CT values were normalized to internal 18S controls and further comparisons were calculated via the ΔΔ*C*_T_ method. Primers for qRT-PCR are listed in Table 1.

### 2.7. Statistical Analysis

*P* values were calculated as indicated in the appropriate figure legends. The average fold change in experiments was calculated using the variable bootstrapping method [28].

## 3. Results

### 3.1. Alphaviruses Produce Distinct Infectious Populations

**Figure 1:**
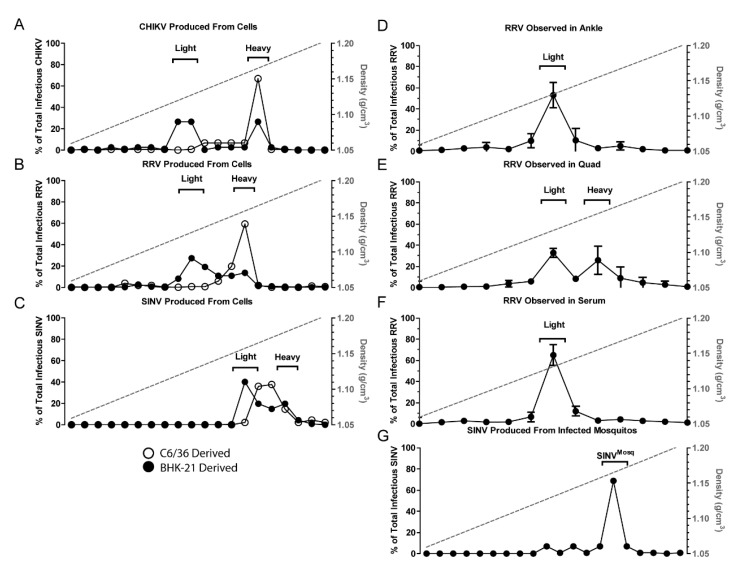
Host type affects virus subpopulations. Purified virus particles were separated into subpopulations by centrifuging in a 15–45% sucrose gradient. Fractions of the gradient were then assayed for infectious units. (**A**–**C**) Virus particles derived from BHK-21 cells (closed circles) exhibit two populations termed Light and Heavy. Particles derived from C6/36 cells (open circles) only exhibit one population; (**D**–**F**) RRV extracted from the indicated mouse tissues one day post-subcutaneous injection; (**G**) SINV extracted from *A. aegypti* mosquitoes one day post-intrathoracic injections. Panel C is adapted from [27]

Initially, we sought to determine whether the production of alphavirus particles with differing densities was observed with other alphavirus species. To determine if other alphaviruses also produced similar infectious particle populations with distinct densities, CHIKV and RRV particles were purified from supernatants of infected BHK-21 cells and exposed to sucrose gradient centrifugation. The gradient was fractionated and the TCID_50_ of each fraction determine to establish the density of infectious particles. Two peaks of infectivity, indicating the presence of multiple particle densities, were observed for CHIKV and RRV, much like SINV (Figure 1). As might be expected, the infectious populations of different virus species differed slightly from one another. Nonetheless, for CHIKV and RRV, the heavy population derived from vertebrate cells migrated at an equivalent density to particles derived from C6/36 cells. This was not observed with SINV where the density of SINV^C6/36^ was found to be between SINV^Light^ and SINV^Heavy^.

To determine if similar and discrete populations of infectious particles were produced in vivo, WT C57BL/6J mice were given subcutaneous injections with 10^3^ plaque forming units of RRV (T48), and samples were taken at one day post-infection (DPI) from various tissues. RRV was used for vertebrate studies as the system is well characterized, and pathology reflective of human disease. Whole mosquitoes were also intrathoracically injected with SINV to determine progeny particle densities. Cellular debris was removed from homogenized tissue samples prior to ultracentrifugation. The virus samples were separated by sucrose gradient centrifugation and fractionated. Interestingly, the analyses of viral particles present in different mouse tissues indicated that the production of heavy and light particles differed in a tissue dependent manner. The infectious RRV particles harvested from ankle joint (bone and cartilage) indicated that the presence of a single population of alphavirus particles was detected, which correlated with the density of RRV^Light^ particles from tissue culture (Figure 1B,D). In contrast, assessment of the viral particles associated with quadriceps muscle tissue revealed that both the RRV^Light^ and RRV^Heavy^ populations were present (Figure 1E), and as shown in Figure 1F, only RRV^Light^ was detected in the serum of infected animals.

Assessments of SINV particles derived from infected whole mosquitoes reaffirmed the particle observations made using tissue culture models. SINV particles extracted from infected mosquitoes consisted of a single population like that of particles derived from C6/36 cells (Figure 1G). Importantly, the relative densities of the C6/36- and mosquito-derived populations were equivalent (1.17 g/cm^3^). 

Collectively, these observations are likely consequential to the transmission of alphaviruses. These data indicate that the particles ingested by the mosquito vector during an infectious blood meal (serum derived virus) will be light particles, whereas as those delivered to naïve vertebrate hosts will be synonymous to the heavy particle species.

### 3.2. Per Os Infections with Viral Subpopulations

**Figure 2:**
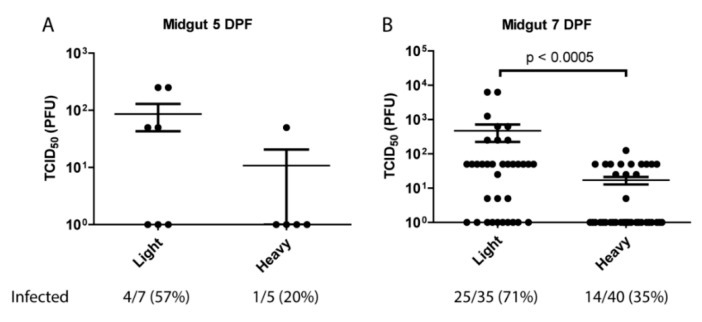
Analysis of particle infectivity in mosquitoes following blood meal. Five to seven-day old female mosquitoes were fed rabbit blood supplemented 1:4 with 1 × 10^8^ particles of either light or heavy SINVMRE3’2J-GFP. Midguts were dissected at (**A**) 5 days or (**B**) 7-days post-infection and virus quantified by TCID_50_ on Vero cells. *P* values were calculated by Mann–Whitney test. All error bars represent standard error of mean (SEM).

The observation that lighter density virus particles were the only observable population in mouse serum indicated that the mosquito midgut may be primarily contacted by light particles following an infectious blood meal. Previous work has shown that in vertebrate cell lines light particles are significantly less infectious than heavy on a particle:pfu basis [28]. To identify the potential impact of this phenomenon on viral transmission, and to determine whether light and heavy particles differed in their capacity to infect mosquitoes, female *Aedes aegypti* HWE mosquitoes were orally infected with equivalent number of SINV MRE3’2J-GFP heavy or light particles. Following infection midguts were isolated, homogenized, and assayed for TCID_50._ As shown in Figure 2, at 5-days post-blood meal there were indications that SINV^Light^ initiated infection more productively than SINV^Heavy^. This difference became far more apparent at 7-days post-blood meal. Virus titers in the midguts of SINV^Light^ infected mosquitoes were significantly higher than those of SINV^Heavy^ fed mosquitoes, with an average difference of ~1.5 log. Additionally, mosquitoes that were fed light particles exhibited a 71% midgut infection rate (MIR), whereas heavy fed mosquitoes exhibited only a 35% MIR. Together, these data indicated that despite being less infectious in vertebrate tissue culture models of infection, SINV^Light^ is more efficient than SINV^Heavy^ in establishing a productive infection of mosquito midgut tissue following a blood meal.

**Figure 3:**
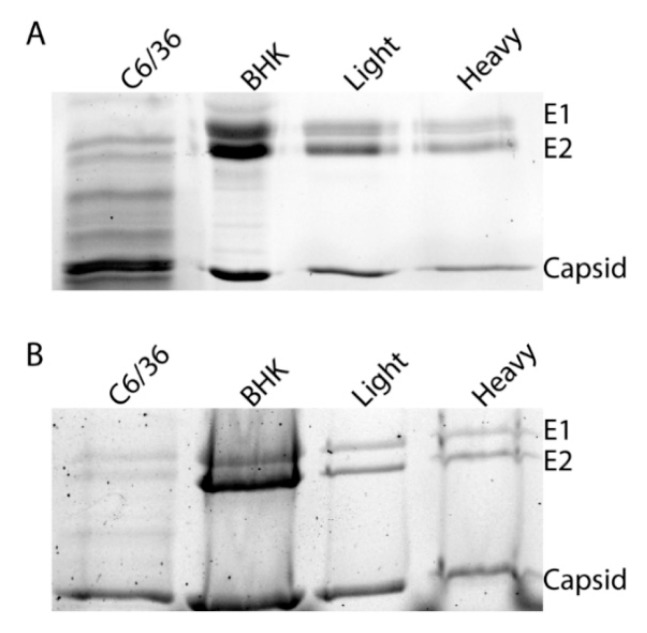
Glycoproteins of SINV subpopulations derived from vertebrate and mosquito cells. Equal number of SINV^Light^ and SINV^Heavy^ virus particles were loaded on a 12% SDS-PAGE and Coomassie staining to assess the migration of E1 and E2 glycoproteins. (**A**) Migration patterns of proteins from particles derived from indicated cell types; (**B**) Migration patterns of proteins from particles following PNGase treatment to remove N-linked glycans.

As it has been previously demonstrated that glycosylation can alter viral transmission, we sought to determine if these differences were due to an obvious difference in glycan processing. To determine if the different infectivities of SINV^Light^ and SINV^Heavy^ were due to different glycosylation patterns, purified virus particles were subjected to SDS-PAGE to examine the migration patterns of the glycoproteins. As expected, the glycoproteins of SINV^C6/36^ and SINV^BHK-21^ exhibited different migration patterns due to the difference in sugar additions (Figure 3A) [33]. Glycoproteins from SINV^Light^ and SINV^Heavy^ comigrated which indicated that there were no obvious differences in glycosylation that could account for altered infectivity in midgut tissue. As a control, virus samples were treated with PNGase F to remove all N-linked oligosaccharides (Figure 3B). This was done to ensure that glycoproteins from the different virus populations possessed no obvious differences that could be masked by the presence of glycan additions.

### 3.3. Bypassing the Midgut Barriers

**Figure 4:**
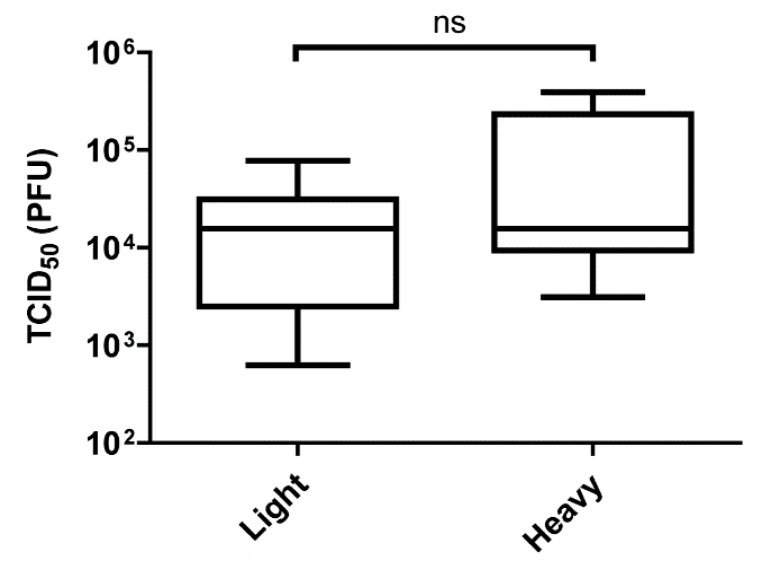
Virus yield of injected SINV in whole mosquitoes. Five to seven-day old female mosquitoes were injected with 5 × 10^5^ particles of MRE3’2J-GFP. The mosquitoes were then snap frozen 2 days post-injection and then homogenized. The homogenate was then serially diluted and added to Vero cells to determine TCID_50_. All error bars represent standard error of mean (SEM).

Having established that differences in mosquito infection and dissemination are observable between infections of SINV^Light^ and SINV^Heavy^, we next sought to determine if the enhanced infectivity of SINV^Light^ is restricted to midgut tissue. Five to seven-day old female mosquitoes were intrathoracically injected with equal numbers of SINV^Light^ and SINV^Heavy^ particles. The TCID_50_ from whole infected mosquitoes was determined two days post-injection. Following intrathoracic injection of the SINV particle species, there was no observable difference in the time it took for GFP signal to be detected [34]. In addition, as shown in Figure 4, no significant difference between SINV^Light^ and SINV^Heavy^ replication in the mosquito following intrathoracic injection was observed indicating that the difference between SINV^Light^ and SINV^Heavy^ infection of mosquitoes is confined to the midgut. These data indicated that following midgut infection and dissemination, any fitness advantage that SINV^Light^ had is lost.

### 3.4. Determining the Midgut Immune Response

**Figure 5:**
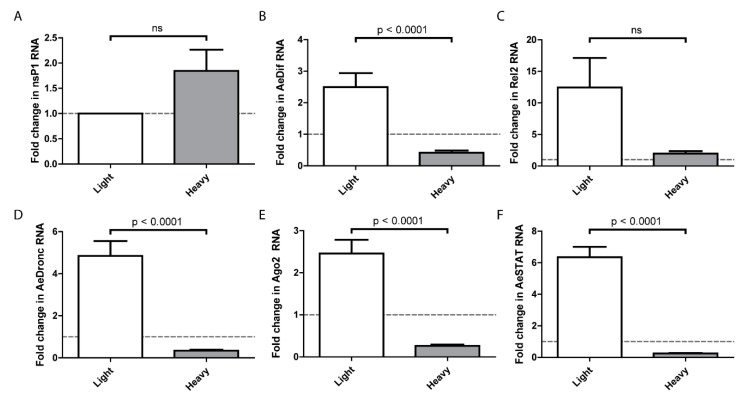
Midgut immune response. Infected midguts were dissected out at 1 DPF and homogenized. RNA was extracted with TRIzol and cDNA was synthesized to quantify RNA levels of (**A**) nsP1 and indicator genes for Toll (**B**), IMD (**C**), JAK-STAT (**D**), apoptosis (**E**), and RNAi (**F**) pathways. *P* values were calculated by Mann–Whitney test. All error bars represent standard error of mean (SEM). The dashed line indicates gene expression levels in mosquitoes following a noninfectious blood meal.

On the basis that SINV^Light^ infects the midgut more efficiently than SINV^Heavy^, we hypothesized that there is a differential host response to the particles, and that the host response to SINV^Light^ facilitated infection. To examine this, the midguts of females were dissected at 24 h after a blood meal containing SINV^Light^ or SINV^Heavy^. Virus replication of SINV^Light^ and SINV^Heavy^, as measured by nsP1 levels, did not indicate any statistically significant differences at one day postinfection (Figure 5A). It should be noted that this contrasts with the data in Figure 2, however, the data in Figure 2 was obtained at 7 days post-infection whereas that in Figure 5 was obtained 1 day post-infection. Expression of genes associated with major known host response pathways were analyzed by qRT-PCR and compared to mock infected midguts (Figure 5B–F) [17,35,36,37,38,39,40]. Unexpectedly, SINV^Light^ infections induced a robust immune response that is significantly higher than SINV^Heavy^ infected midguts. Following SINV^Light^ infection, the genes representing the Toll and JAK-STAT pathways exhibited significantly higher expression levels compared to SINV^Heavy^ infection. Similar trends were observed for the apoptotic pathway, as indicated by *dronc* RNA levels, and for the RNAi pathway, as indicated by *ago2* RNA levels. As indicated by *rel2* expression, the IMD pathway was not induced to a statistically significant level following SINV^Light^ infection compared to SINV^Heavy^ infection. Interestingly, the Toll, JAK-STAT, apoptosis, and RNAi pathways showed a suppression of the immune response in SINV^Heavy^ infected midguts whereas the IMD pathway did not show the same pattern (Figure 5C).

## 4. Discussion

We previously reported that SINV infection of vertebrate cell lines yields two populations of infectious particles separable based on density. Mass spectrometry and qRT-PCR analysis of RNA in the particle revealed that one subpopulation of virus particles had incorporated host derived ribosomal components (HDRCs). In contrast, a single population of particles was derived from mosquito cells, which contained HDRCs and strongly resembled the heavy particle population derived from vertebrate cells. The presence of the HDRCs in particles correlated with increased infectivity in vertebrate cell lines [27]. While the enhanced infectivity of SINV^Heavy^ and SINV^C6/36^ particles in vertebrate cell lines was interesting to note, the greater abundance of the less infectious SINV^Light^ produced from vertebrate cells was somewhat puzzling. Here, we have shown that the production of two distinct particle types was not limited to SINV, as both CHIKV and RRV produced heavy and light particles in vertebrate cells, and for these viruses the mosquito cell derived particles corresponded in density to the vertebrate derived heavy particles (Figure 1). It was also apparent that SINV particles were, in general, denser than the CHIKV and RRV particles (Figure 1C). Additionally, the SINV^C6/36^ particles displayed an intermediate density between that of SINV^Light^ and SINV^Heavy^ whereas CHIKV^C6/36^ and RRV^C6/36^ had the same densities as their corresponding vertebrate derived heavy subpopulations. It is possible that the different particle densities are due to the genetic differences between the three species tested. Further exploration of these differences in particle density is necessary as other viruses in the WEEV complex will need to be compared to SINV to determine if this is a characteristic common to viruses in the WEEV complex. However, the fundamental finding that each of the alphavirus species examined produced heavy and light particles in vertebrate cells indicates that this is the case for all alphavirus species, and that findings on the biological relevance of the different subpopulations can be applied across the genus.

A major contribution of this study is the examination of particle types produced by RRV infected mice. As shown above, analyses of infected joint, muscle, and serum revealed differences in the proportions of heavy and light particles produced in different tissues (Figure 1D–F). In muscle tissue (Figure 1E), infectious particles were found in almost equal proportions of heavy and light fractions, however, in serum, infectious particles were found only in the light fraction (Figure 1F). While it is currently unclear why different tissues produce different proportions of the particle types, the implication is that the sequestration of HDRCs by assembling virus particles is significantly influenced by the host cell type. Given that the presence or absence of the HDRCs has a significant impact on infection in vertebrate cells, the production of different particle types is likely significant for pathogenesis and warrants deeper investigation [27].

The other major implication of the observation that only light particles were detectable in the serum of infected animals was that a feeding mosquito would only be exposed to light particles. Previous work had shown that SINV^Heavy^ particles were far more infectious in vertebrate cells than SINV^Light^, with most of the light particles being noninfectious (particle to p.f.u. ratio of approximately 4:1 versus 80:1, respectively) [27]. When the infectivity of SINV^Light^ was examined in mosquitoes via per os infections, it was apparent that SINV^Light^ infections exhibited a significantly higher viral load in the midgut than SINV^Heavy^ infections at 7 DPI (Figure 2). Electron microscopy and SDS-PAGE analysis of glycoproteins (Figure 3) has indicated no obvious glycosylation differences between SINV^Light^ and SINV^Heavy^ particles [27]. Therefore, it appears that the absence of the HDRCs correlates with increased infection of mosquito midguts. The apparent advantage in infecting the mosquito displayed by SINV^Light^ was lost when the midgut was bypassed by intrathoracic injection of virus (Figure 4). However, in future work it would be of interest to know whether midgut tissue is preferentially infected by SINV^Light^ following intrathoracic infection. Such studies would help determine whether the ability of SINV^Light^ to infect midgut is tissue dependent or delivery dependent. When virus from infected mosquitoes was analyzed, a single population of particles was found that was denser than SINV^Light^ and comigrated in gradients with C6/36 cell-derived virus previously shown to contain HDRCs (Figure 1G) [27]. These data show that, while light particles may initiate infection in the mosquito midgut, virus produced in the mosquito is denser and contains HDRCs.

These observations together imply that initial infection by SINV^Light^ is either initially more efficient in terms of virus production, or leads to a response in the midgut that facilitates subsequent virus infection and replication. The means by which SINV^Light^ infection is promoted and/or SINV^Heavy^ inhibited during midgut infection requires further investigation. However, it was observed that the midgut expression of immunity genes at 1 DPI in response to the two particles types was significantly different despite similar nsP1 levels (Figure 5). We examined expression levels of the primary transcription factors associated with the Toll (*dif*), IMD (*rel2*), and JAK-STAT (*stat*) pathways, as well as siRNA (*ago2*) and apoptosis (*dronc*) associated genes. In all cases, except *rel2*, gene expression was elevated following infection with SINV^Light^ and decreased following infection with SINV^Heavy^ compared to a mock infected control. We hypothesized that this exacerbated response to SINV^Light^ infection facilitated midgut infection. Particularly with apoptosis related genes, previous work has shown that an enhanced response can facilitate midgut escape [40]. Our future work will focus on establishing the molecular patterns recognized by the host cell that distinguish SINV^Light^ from SINV^Heavy^. In previous work we have shown that the presence of HDRCs correlates with the extent to which the viral genomic RNA is capped; SINV^Heavy^ particles contain more capped RNA, whereas SINV^Light^ particles have more noncapped genome packaged [40]. Noncapped RNA is known to function as a trigger for the immune response in numerous systems and may do so in this case [41,42,43,44]. Thus, the activation of the immune response to noncapped RNA present in SINV^Light^ particles may, counterintuitively, aid with midgut infection. However, it is important to recognize that the SINV^Light^ immune trigger would be transient, as following infection, virus produced would contain HDRCs, and, based on prior results, more capped RNA [41]. It is also currently difficult to reconcile the stimulation of expression of RNAi associated genes by SINV^Light^ with enhanced infection. Previous work with alphaviruses has shown the siRNA pathway to be antiviral, therefore it is not clear how increased expression of *ago2* could lead to more efficient establishment of infection [7]. One possibility is that antagonistic responses to infection are occurring, and that a proinfection response is overriding the antiviral effect of the RNAi pathway.

Together, these observations show a correspondence with the transmission cycle. Infected vertebrate hosts produce two distinct subpopulations of virus, but only one, SINV^Light^, is present in the serum and therefore ingested by the mosquito. SINV^Light^ efficiently infects midgut epithelium establishing infection. Following successive rounds of replication, the progeny produced shifts to a homogenous, heavy population to disseminate to other tissues for transmission. This heavy mosquito virus is highly infectious in vertebrate cells, and can therefore efficiently establish infection in the vertebrate host following an infectious bite. The current data and previously published reports show that nonvirally encoded, virus particle cargo regulates the virus infection and transmission cycle [27,41].

## Figures and Tables

**Table 1:** Primer Sequences.

Primer	Sequence
18s F	CGAAAGTTAGAGGTTCGAAGGCGA
18s R	CCGTGTTGAGTCAAATTAAGCCGC
nsP1 F	AAGGATCTCCGGACCGTA
nsP1 R	AACATGAACTGGGTGTCGAAG
AeDif F	ACAAACGTCTCCCTACAATG
AeDif R	ACTGATTCTGGAACTGTTGG
Rel2 F	CAAGAACAGGAAGAGAAC
Rel2 R	CCTCCACTCTATTACAGC
AeDronc F	AAAGAACTGAAGCAGTCCAG
AeDronc R	CGTATAGGACGGAATTATCG
Ago2 F	ACCGAATATCACCTTCATTG
Ago2 R	TAACGGTCAACGATAGTTCC
AeSTAT F	GTTTCGATCATTTCAGAAGC
AeSTAT R	CTTCGTGGTTTCGTTGTACT
